# Primary intracranial plasmablastic lymphoma with intradural extramedullary metastasis: a case report

**DOI:** 10.1186/s12883-025-04409-9

**Published:** 2025-09-29

**Authors:** Zhuoru Jiang, Zhengyang Zhu, Zhennan Tao, Fengnan Niu, Xin Zhang, Bing Zhang

**Affiliations:** 1 Department of Radiology, Nanjing Drum Tower Hospital, Affiliated Hospital of Medical School, Nanjing University, Nanjing, 210009 China; 2 Institute of Medical Imaging and Artificial Intelligence, Nanjing University, Nanjing, 210093 China; 3 Medical Imaging Center, Department of Radiology, Nanjing Drum Tower Hospital, Affiliated Hospital of Medical School, Nanjing University, Nanjing, 210009 China; 4 Department of Neurosurgery, Nanjing Drum Tower Hospital, Affiliated Hospital of Medical School, Nanjing University, Nanjing, 210009 China; 5 Department of Pathology, Nanjing Drum Tower Hospital, Affiliated Hospital of Medical School, Nanjing University, Nanjing, 210009 China

**Keywords:** Plasmablastic lymphoma, Magnetic resonance imaging, Case report

## Abstract

**Background:**

Primary central nervous system plasmablastic lymphoma (PCNSPBL) represents an exceptionally rare and aggressive subtype of diffuse large B-cell lymphoma (DLBCL), characterized by its distinct immunophenotypic profile and predilection for immunocompromised individuals. Accurate preoperative diagnosis remains challenging due to the nonspecific radiological features observed on conventional magnetic resonance imaging (MRI), necessitating comprehensive histopathological evaluation for definitive diagnosis. While intracranial involvement of PBL has been documented in the literature, intraspinal metastasis of this malignancy has not been previously reported. This study presents a novel case of PCNSPBL with concurrent spinal cord metastasis in an elderly male patient, with a detailed analysis of advanced neuroimaging characteristics that may aid in the diagnostic evaluation of this rare entity.

**Case presentation:**

A 52-year-old male patient presented to our institution with left-sided limb weakness. Laboratory investigations revealed positive Epstein-Barr virus-encoded small RNA (EBER). Conventional MRI demonstrated a mass lesion with a clear anatomical relationship to the inferior horn of the right lateral ventricle, presenting as compression and stenosis of the right ventricle. Due to the mass effect, the patient underwent surgical resection of the right temporal lobe lesion, with histopathological examination confirming the diagnosis of plasmablastic lymphoma.

**Conclusions:**

This report represents the first documented case of PCNSPBL with intradural extramedullary metastasis, underscoring the diagnostic challenges associated with this rare malignancy. Advanced neuroimaging techniques, including functional MRI, serve as valuable complements to conventional MRI in the diagnostic workup. Early and accurate diagnosis of PBL is critical for timely intervention, and further studies are warranted to establish standardized imaging protocols for this rare entity.

**Supplementary Information:**

The online version contains supplementary material available at 10.1186/s12883-025-04409-9.

## Introduction

Plasmablastic lymphoma (PBL), a rare and clinically aggressive variant of diffuse large B-cell lymphoma (DLBCL), poses significant diagnostic challenges due to its heterogeneous clinical presentations and nonspecific pathological features [1]. Although PBL predominantly arises in the oral cavity, emerging evidence has documented its extraoral manifestations across multiple anatomical systems, including the sinonasal tract (maxillary sinus, nasopharynx), gastrointestinal system (stomach, small bowel, anus), respiratory system (lung), integumentary system (skin), soft tissues, and even rare sites such as the heart and spermatic cord [2–5]. Of particular clinical significance is primary central nervous system PBL (PCNSPBL), an exceptionally rare entity that accounts for less than 1% of all CNS lymphomas [6, 7]. The diagnostic paradigm for PCNSPBL requires a multidisciplinary approach, integrating clinical suspicion with histopathological confirmation, while recognizing the limitations of conventional neuroimaging findings that often demonstrate nonspecific characteristics, potentially leading to diagnostic ambiguity or oversight [8, 9]. Recent advances in neuroimaging modalities, including advanced MRI techniques and metabolic imaging, have provided novel insights into the radiologic features of CNS lymphomas [10–12]offering improved diagnostic accuracy in differentiating PBL from other intracranial pathologies. In this context, we present a comprehensive neuroimaging evaluation of a rare case of intracranial plasmablastic lymphoma with intradural extramedullary metastasis, aiming to contribute to the growing body of literature on the radiologic-pathologic correlation of this rare disease entity.

## Case presentation

A 52-year-old Man experienced left Limbs weakness for over 1 month. Magnetic Resonance Imaging (MRI) performed at the local hospital suggested abnormal signals in the right side of the brainstem, the right basal ganglia and the temporal lobe. Neurological physical examination revealed that the muscle strength of the left Limbs was graded as 4, while the right Limbs exhibited grade 5 strength. Serological testing confirmed negative HIV antibody status.

**Fig. 1:**
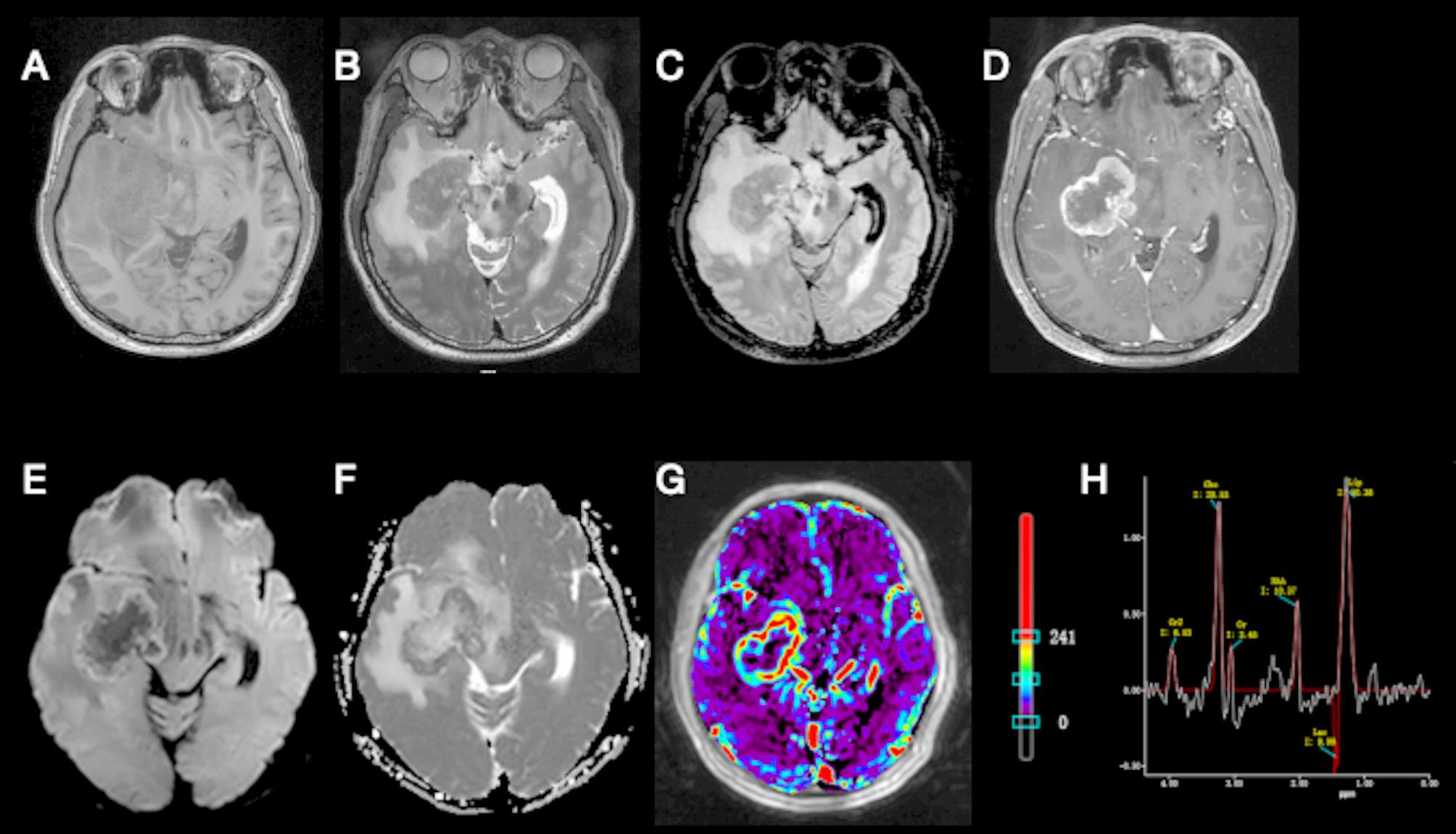
The initial MRI brain images before the first surgery. On MRI, a well-defined Mass measuring approximately 44×47×39 mm is observed in the right basal ganglia, insulotemporal lobe, midbrain, and brainstem. The lesion demonstrates isointense signal on T_1_-weighted imaging and isointense signal on T_2_-weighted imaging. DWI reveals mild hyperintensity along the lesion margins with corresponding reduced signal on ADC maps, suggesting restricted diffusion. Surrounding the lesion, extensive peripheral edema is evident, appearing as hypointense on T_1_-weighted images and hyperintense on T_2_-weighted images. Post-contrast sequences show marked heterogeneous enhancement within the lesion. Additionally, bilateral periventricular white matter abnormalities are noted, displaying mildly prolonged T_1_ and T_2_ signals with hyperintensity on FLAIR sequences.(A) Axial T_1_W sequence, pre-contrast; (B) axial T_2_W sequence; (C) axial T_2_-FLAIR sequence, pre-contrast; (D) gadolinium-enhanced MR T_1_-weighted image; (E) axial diffusion weighted imaging sequence; (F) apparent diffusion coefficient image; (G) transfer constant image; (H) magnetic resonance spectroscopy image (TR/TE=2000/144 milliseconds). Cho = 28.53, Lip = 50.36, Cr = 3.45.

Conventional MRI, dynamic contrast enhanced MRI (DCE-MRI) (Table 1) and MR spectroscopy were obtained. Brain MRI showed obvious enhancement with cystic necrosis and large area of peritumor edema. Diffusion weighted imaging (Fig. 1) showed restricted diffusion, indicating high cell density of the tumor. MR spectroscopy (Fig. 1) revealed a slight increase choline (Cho) peak and a slight decrease in the peaks of creatine (Cr) and N-acetylaspartate (NAA). There were abnormal peaks of lipid (Lip) and lactate (Lac). The Cho/Cr ratio was 8.27 while Cho/NAA ratio was 2.75.

**Table 1:** Cerebral hemodynamic parameters of the ROI of the lesion and normal control

Before the First Surgery		
Parameter	ROI of the legion	ROI of normal control
Ktrans (10^−3^/min)	182.7	33.5
Kep (10^−3^/min)	525.8	2639.7
Ve (10^−3^)	350.4	19.1
After the First Surgery		
Parameter	ROI of the legion	ROI of normal control
Ktrans (10^−3^/min)	84.1	27
Kep (10^−3^/min)	448.5	1634.9
Ve (10^−3^)	317	37

Considering the size and enhancing lesions in the right basal ganglia region, insular and temporal lobes, midbrain and brainstem, accompanied by extensive edema of the surrounding brain tissue, with dissemination in the right cerebellopontine angle region, after excluding contraindications, the patient underwent right temporal lobe tumor resection with dural repair and cranioplasty. The pathology of the resected tumor indicated plasmablastic lymphoma, but the patient refused to take chemotherapy.

**Fig. 2:**
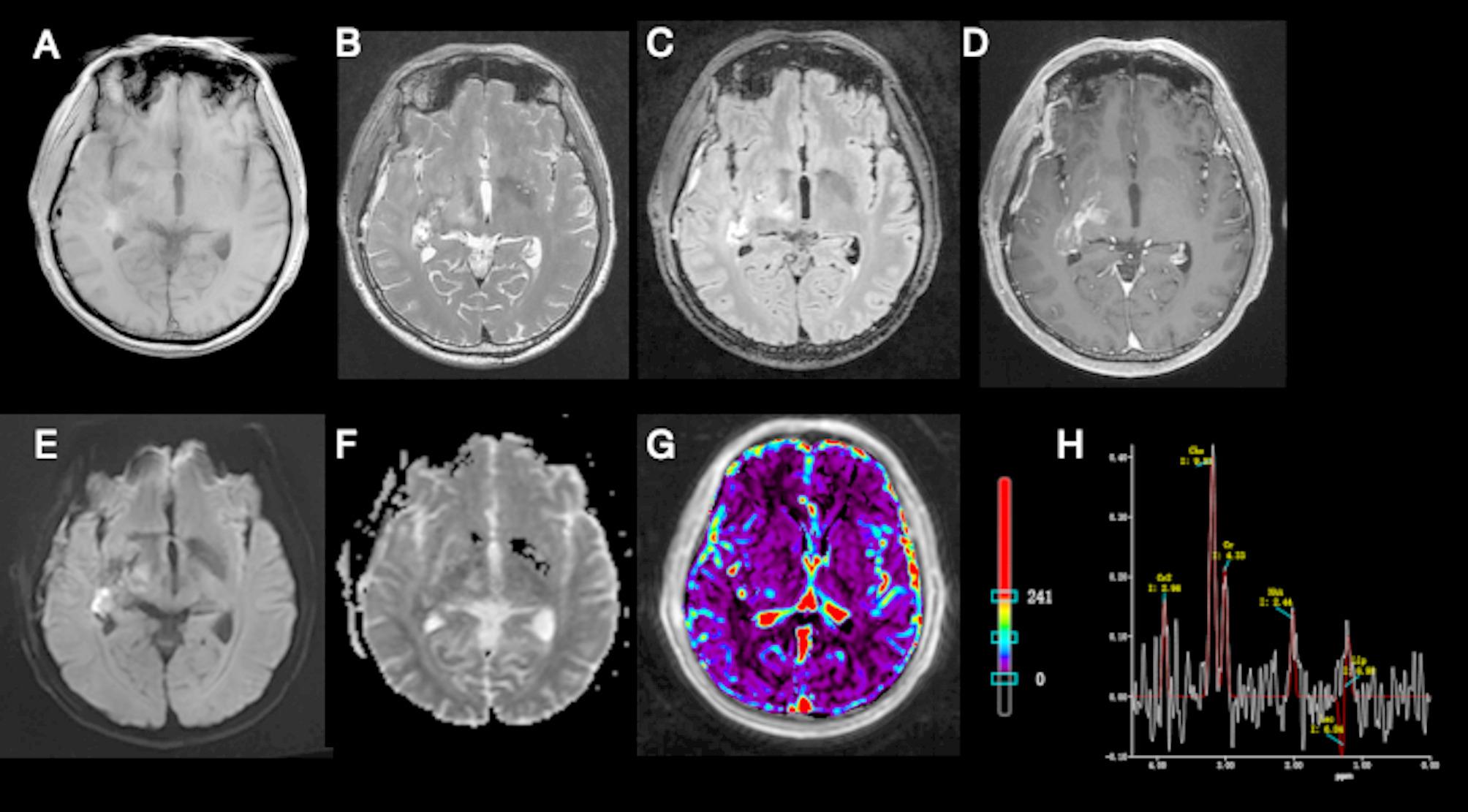
The MRI brain images after the first surgery. (**A**) Axial T_1_W sequence, pre-contrast; (**B**) axial T_2_W sequence, pre-contrast; (**C**) axial T_2_-FLAIR sequence, pre-contrast; (**D**) gadolinium-enhanced MR T_1_-weighted image; (**E**) axial diffusion weighted imaging sequence; (**F**) apparent diffusion coefficient image; (**G**) transfer constant image; (**H**) magnetic resonance spectroscopy image. Cho = 9.53, Lip = 6.98, Cr = 4.33

As is shown in Fig. 2, postoperative MRI demonstrates an irregular surgical cavity in the right temporal lobe exhibiting heterogeneous signal intensity with predominant T_1_ hypointensity and T_2_ hyperintensity, surrounded by FLAIR hyperintense parenchyma. Scattered punctate DWI hyperintensities are noted within the cavity. The cavity Margins show irregular contrast enhancement. Adjacent dural thickening with Linear enhancement is observed. New lesions are identified in the right cerebellopontine angle and right thalamus. The cerebellopontine angle lesion presents as a 6-mm ovoid focus demonstrating T_1_ hypointensity, T_2_ hyperintensity, DWI hyperintensity, and absence of contrast enhancement. Similarly, the thalamic lesion is a 5 mm rounded focus exhibiting identical signal characteristics: T_1_ hypointensity/T_2_ hyperintensity/DWI hyperintensity without enhancement. Additional findings include a 3 mm punctate left thalamic focus with T_1_/T_2_ prolongation but no DWI abnormality or enhancement, and bilateral frontoparietal white Matter scattered 24 mm foci displaying mild T_1_ hypointensity/T_2_ hyperintensity, FLAIR hyperintensity, and DWI isointensity.

**Fig. 3:**
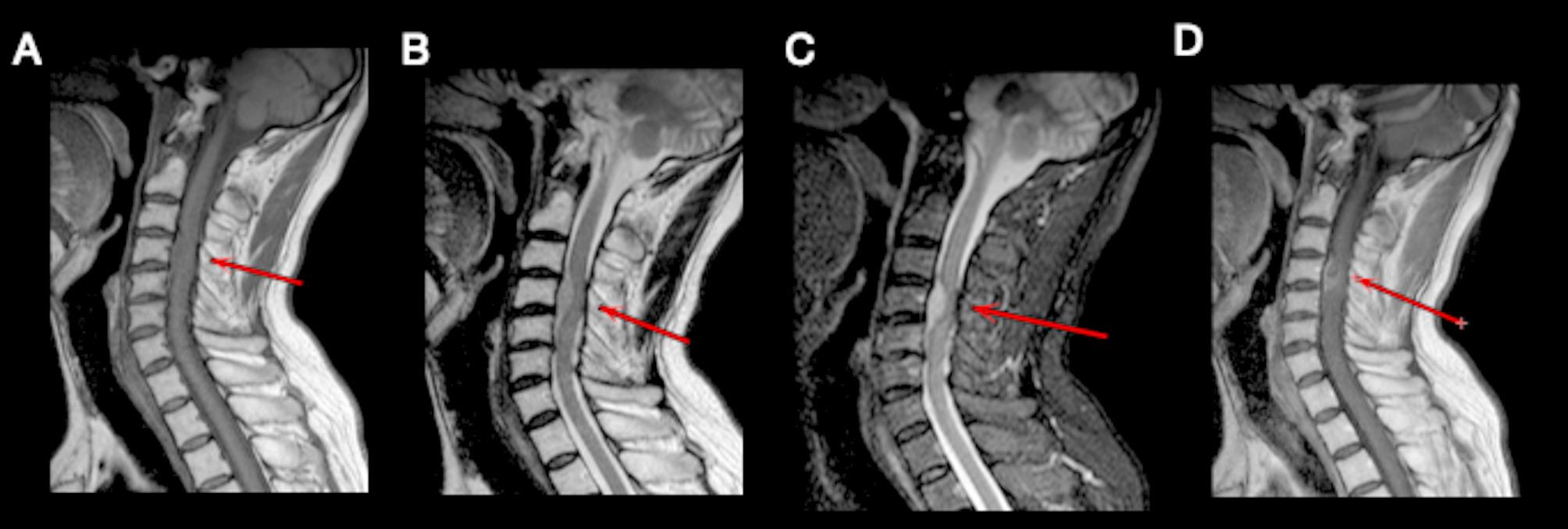
Cervical spine MRI images. (A) Sagittal T_1_W sequence, pre-contrast; (B) sagittal T_2_W sequence; (C) sagittal T_2_-STIR sequence; (D) gadoliniuma focal enhancing lesion at C4 level.

Five months after the first surgery (Table 1), the patient was admitted again for neck discomfort. MRI of the cervical spine demonstrated an intradural extramedullary mass at the C4 vertebral level, measuring approximately 18 mm×10 mm×22 mm, with clear enhancement on contrast imaging and a dural tail sign, suggesting spinal cord compression (Fig. 3). Cytology and special staining for hematologic malignancies revealed lymphocytosis (96%). He underwent the second surgery, resection of intraspinal schwannoma with C3-C5 laminoplasty and spinal dural repair. Immunophenotyping of the lesions within the spinal canal revealed a population of cells positive for PAX5, Bcl2 (80%++), MUM1 and CD79a; it was negative for Olig-2, GFAP, CK, CD3, CD20, CD10, Bcl6, Kappa and Lambda. Clonal immunoglobulin gene rearrangements (IGH-DH, IGκ-V/in) were detected. The in situ hybridization experiment suggested positive for EBER.

The patient underwent four cycles of intravenous chemotherapy with Lenalidomide + CHOP after the second surgery. However, in preparing autologous stem cell transplantation, he underwent seizures, urinary and fecal incontinence, altered mental status, nausea, and vomiting. then he became bedridden. The patient passed away shortly thereafter.

## Discussion

Plasmablastic lymphoma (PBL), a rare and aggressive subtype of diffuse large B-cell lymphoma [13]has been documented in various anatomical sites, including the brain and epidural space, with spinal cord compression reported in isolated cases [7, 14–16]. While PBL predominantly affects immunocompromised individuals, particularly those with HIV infection, and frequently involves extranodal sites such as the oral cavity, its pathogenesis remains incompletely elucidated [17–19]. The disease is characterized by a distinct immunophenotypic profile, marked by the loss of conventional B-cell markers (e.g., CD20) and the expression of plasma cell-associated antigens (e.g., CD138, MUM1) [20–22]. This unique immunophenotype, coupled with its aggressive biological behavior, contributes to significant diagnostic and therapeutic challenges.

Central nervous system (CNS) involvement by PBL, though rare, presents with a spectrum of nonspecific clinical manifestations, including cognitive impairment, focal neurological deficits, and symptoms of elevated intracranial pressure [15]. Radiologically, cerebral PBL typically manifests as solitary or multifocal enhancing lesions with perilesional edema, often involving periventricular regions and deep gray matter structures. Urrego et al. [23] reported a patient with PCNSPBL, an axial T_1_-weighted contrast-enhancing MRI showed a solid heterogeneous area of patterned enhancement with scalloped peripheral low signal enhancement, higher signal enhancement along the juxtaposed white matter. However, the absence of pathognomonic imaging features frequently leads to diagnostic ambiguity, necessitating histopathological confirmation. Notably, our case demonstrated cystic necrosis on MRI, a feature that deviates from the more common solid appearance of CNS lymphomas and may mimic high-grade gliomas, further complicating the diagnostic process.

Spinal lymphoma occurs more in predominantly non-Hodgkin lymphoma, especially diffuse large B-cell subtype than in Hodgkin lymphoma, and typically affects middle-aged to older adults with progressive myelopathy. Most cases represent secondary leptomeningeal dissemination rather than isolated spinal disease. Imaging reveals poorly defined intramedullary masses: T1-isointense, T2-hyperintense, with solid homogeneous enhancement, though gadolinium uptake varies [24]. Intradural extramedullary metastasis by primary CNS lymphoma is exceptionally rare and presents distinct diagnostic challenges. Patients often exhibit myelopathic symptoms, including sensory deficits, motor weakness, and sphincter dysfunction. Imaging findings typically include intramedullary lesions with variable enhancement patterns and associated spinal cord swelling. In the report of Gao et al. [16]a discus-like lesion was located posterior to the spinal cord and showed an isointense signal on T_1_- and T_2_-weighted images. After injection of an intravenous contrast agent, the lesion was homogeneously enhanced with a typical mouse tail sign. Ella et al. [25] reported that the PBL extended through the exit foramina with a small enhancing paraspinal component. Differential diagnoses encompass a broad spectrum of intramedullary pathologies, including astrocytoma, ependymoma, and metastatic lesions. The presence of multifocal involvement, rapid progression, and systemic symptoms may raise clinical suspicion for PBL, though definitive diagnosis requires histopathological confirmation. In our case, the intradural extramedullary mass was suspected to represent secondary lymphomatous involvement, marking the first reported instance of CNS PBL with intradural extramedullary metastasis.

Advanced neuroimaging techniques, including diffusion-weighted imaging (DWI), dynamic contrast-enhanced MRI (DCE-MRI), and magnetic resonance spectroscopy (MRS), have significantly enhanced the diagnostic evaluation of CNS PBL. DCE-MRI provides insights into microvascular physiology [26]while MRS offers metabolic profiling, revealing alterations in key metabolites such as choline (Cho), creatine (Cr), and N-acetylaspartate (NAA) [27]. CNS lymphomas typically exhibit a characteristic spectroscopic pattern: elevated Cho, decreased NAA, and increased lipid peaks. Crucially, the lipid elevation occurs primarily within the solid, non-necrotic tumor component—a key feature distinguishing them from gliomas, where lipid peaks are usually associated with necrosis [28]. In our case, MRS demonstrated a mild increase in Cho and decreased Cr and NAA (Cho/Cr = 8.27; Cho/NAA = 2.75), consistent with cellular proliferation and neuronal injury.

There are other advanced neuroimaging techniques not used in this case, but necessary in diagnosing CNS PBL. Perfusion-weighted MRI techniques measure microcirculatory parameters, including cerebral blood volume (CBV), cerebral blood flow (CBF), and mean transit time (MTT). A diagnostically significant finding is that lymphomatous lesions consistently show low CBV. This is reflected in a low maximum relative CBV (rCBV) ratio when comparing the tumor to contralateral normal brain tissue, distinguishing lymphoma from other brain tumors which typically have higher rCBV [29].

While advanced imaging including perfusion and spectroscopy was utilized, whole-brain volumetric analysis and diffusion tensor imaging (DTI) tractography were not performed in this case. Future studies would benefit from incorporating these techniques to quantitatively evaluate tumor burden and white matter integrity, particularly for assessing treatment response and predicting neurological outcomes in CNS lymphoma.

## Conclusion

PCNSPBL represents a rare, diagnostically challenging malignancy with nonspecific presentations, where diagnostic delays stem from lacking definitive imaging biomarkers and low clinical awareness. While advanced neuroimaging aids differentiation from other CNS pathologies, histopathological confirmation remains the gold standard. This first-reported case of CNS PBL with intradural extramedullary metastasis underscores the necessity for multidisciplinary diagnostic approaches and heightened suspicion in immunocompromised patients presenting with atypical CNS lesions. Future research must prioritize identifying specific imaging biomarkers and developing standardized diagnostic protocols to improve management of this aggressive tumor.

## Supplementary Information

Below is the link to the electronic supplementary material.

Supplementary Material 1

## Data Availability

No datasets were generated or analysed during the current study.
