# Supplementary material for: Primary intracranial plasmablastic lymphoma with intradural extramedullary metastasis: a case report
Source: BMC Neurol. 2025 Sep 29;25:394. doi: 10.1186/s12883-025-04409-9 (PMC12481800; doi:10.1186/s12883-025-04409-9)
Supplement: Supplementary file 1 — Supplementary Material 1 [file 12883_2025_4409_MOESM1_ESM.docx]

**Primary Brain Plasmablastic Lymphoma with Spinal Cord Metastasis: a Case Report**

Zhuoru Jiang^1,2,3^*, Zhengyang Zhu^1,2,3^*, Zhennan Tao^4^, Fengnan Niu^5^, Xin Zhang^1,2,3#^, Bing Zhang^1,2,3^

1 Department of Radiology, Nanjing Drum Tower Hospital, Affiliated Hospital of Medical School, Nanjing University

2 Institute of Medical Imaging and Artificial Intelligence, Nanjing University

3 Medical Imaging Center, Department of Radiology, Nanjing Drum Tower Hospital, Affiliated Hospital of Medical School, Nanjing University

4 Department of Neurosurgery, Nanjing Drum Tower Hospital, Affiliated Hospital of Medical School, Nanjing University

5 Department of Pathology, Nanjing Drum Tower Hospital, Affiliated Hospital of Medical School, Nanjing University

*These authors contributed equally to this work and share first authorship。

**#Correspondence to**:

Xin Zhang: [neuro_zx@163.com](mailto:neuro_zx@163.com)

Supplementary Data

All the preoperative MRI date were acquired using a 3.0T MRI scanner (uMR790, United Imaging Healthcare, Shanghai, China) with a 32-channel phased-array head coil. Conventional MRI examinations included 3D T1WI pre- and post- the injection of gadolinium-based contrast agent (repetition time [TR]/ echo time [TE]=7.9/3.1 milliseconds; inversion time [TI]=810 milliseconds; flip angle [FA]=10◦; matrix=256 × 256; field of view [FOV]=256 × 232 mm2; slice thickness=1 mm), 3D T2WI (TR/TE=2200/606.36 milliseconds; TI=1519 milliseconds; FA: from 19° to 150°; matrix=256 × 256; FOV=256 × 232 mm2; slice thickness=1 mm) and 3D FLAIR (TR/TE=4800/428.04 milliseconds; TI=1519 milliseconds; FA: from 21° to 150°; matrix=240 × 240; FOV=256 × 232 mm2; slice thickness=1mm).

Axial DCE-MRI acquisition was performed using dynamic scan of a T1-gradient echo sequence and setting the following parameters: TR/TE=3.47/1.9 milliseconds; FA=13°; matrix=160 × 160; FOV=240 × 220 mm2; slice thickness=5 mm. Pre-contrast images with multiple FA 5, 10 and 15° were acquired for the T1 maps. Then the contrast agent (Gadovist, 1 mmol/mL, Bayer Healthcare, Berlin, Germany) was administered (0.1mmol/kg of bodyweight) through the antecubital vein via a power injector at a rate of 2 mL/s. A series of 1800 images at 90 dynamic phases for 20 axial sections were obtained with a temporal resolution of 4 seconds for each dynamic phase.

DCE metrics were calculated using United imaging software workstation.

The following DCE parameters were analyzed based on the Tofts model: Ktrans, Kep, Ve and iAUC. Ktrans refers to the volume transfer constant, signifying the flow of gadolinium from the blood plasma into the extravascular extracellular space (EES); Kep denotes the time constant of gadolinium reflux from the EES back into the vascular system; Ve represents the EES volume per unit tissue volume and iAUC illustrates to the initial area under the time-concentration curve for the first 60 seconds.
